# Gradient doping of phosphorus in Fe_2_O_3_ nanoarray photoanodes for enhanced charge separation†
†Electronic supplementary information (ESI) available. See DOI: 10.1039/c6sc03707k



**DOI:** 10.1039/c6sc03707k

**Published:** 2016-10-03

**Authors:** Zhibin Luo, Chengcheng Li, Shanshan Liu, Tuo Wang, Jinlong Gong

**Affiliations:** a Key Laboratory for Green Chemical Technology of Ministry of Education , School of Chemical Engineering and Technology , Tianjin University , Tianjin 300350 , China . Email: jlgong@tju.edu.cn ; Email: wangtuo@tju.edu.cn; b Collaborative Innovation Center of Chemical Science and Engineering , Tianjin 300350 , China

## Abstract

Hematite (α-Fe_2_O_3_) is a promising candidate for solar-to-hydrogen energy conversion. However, the low carrier mobility and extremely high charge recombination rate limit the practical application of hematite in solar water splitting. This paper describes the fabrication of a Fe_2_O_3_ photoanode with gradient incorporation of phosphorus (P) employing a facile dipping and annealing method to improve the charge separation for enhanced photoelectrochemical water oxidation. This gradient P incorporation increases the width of band bending over a large region in Fe_2_O_3_, which is crucial for promoting the charge separation efficiency in the bulk. Although both gradient and homogeneous P-incorporated Fe_2_O_3_ samples exhibit similar electrical conductivity, the Fe_2_O_3_ electrode with a gradient P concentration presents an additional charge separation effect. A photocurrent of ∼1.48 mA cm^–2^ is obtained at 1.23 V *vs*. reversible hydrogen electrode (*vs.* RHE) under air mass 1.5G illumination. Additionally, the H_2_O oxidation kinetics of Fe_2_O_3_ with gradient P incorporation was further improved upon loading cobalt phosphate as cocatalyst, reaching a photocurrent of ∼2.0 mA cm^–2^ at 1.23 V *vs.* RHE.

## Abstract 2

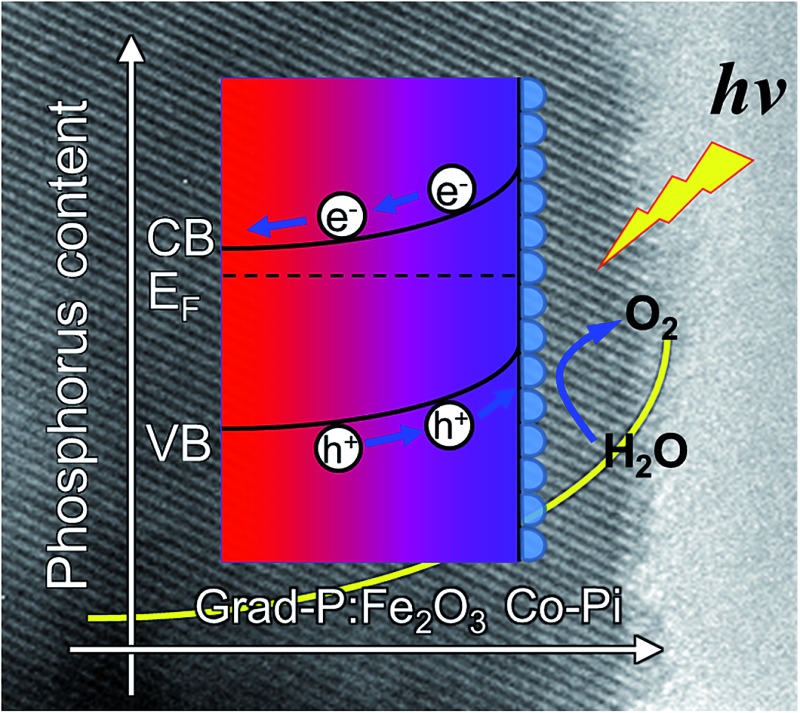
Highly-oriented Fe_2_O_3_ nanoarrays with a gradient phosphorus concentration result in enhanced charge separation in the bulk for photoelectrochemical water oxidation.

## Introduction

The rocketing demand for energy supplies has depleted fossil fuels and has caused global environmental problems.^1,2^ Since the initial work on light-driven water splitting over TiO_2_, photoelectrochemical (PEC) water splitting harvesting sunlight has been widely accepted as one of the most promising routes for hydrogen production.^3,4^ Hematite (α-Fe_2_O_3_) is a nontoxic and photostable n-type semiconductor with an indirect bandgap of about 2.1 eV,^5^ which can theoretically allow the utilization of approximately 40% of the solar spectrum. However, relatively poor conductivity, rapid electron–hole recombination rates, short hole diffusion length (2–4 nm) and sluggish oxygen-evolution kinetics are intrinsic limitations of hematite for effective solar water splitting.^6^ Various strategies have been proposed to address these obstacles, including element doping, nanostructure engineering, and surface modifications (such as depositing passivation layers and oxygen-evolution cocatalysts).^7^

Recently, progress has been achieved to address the limitations of hematite through improving solar conversion efficiency *via* several strategies. Grätzel and co-workers fabricated Si-doped dendritic hematite films with an outstanding PEC performance.^5^ Sn-doped hematite nanowires synthesized by Li and co-workers also showed a remarkable photocurrent density.^8^ Sivula *et al.* demonstrated that mesoporous hematite yielded impressive water-oxidation photocurrents.^9^ Phosphorus (P) doped hematite reported by Chen and co-workers exhibited an extraordinary PEC performance owing to the enhanced electron mobility.^10^ Wang and colleagues attained a recorded turn-on voltage of 0.45 V *versus* reversible hydrogen electrode (*vs.* RHE) with NiFeO_*x*_ cocatalyst.^11^ Lee and co-workers prepared a unique single-crystalline “wormlike” morphological hematite photoanode modified by platinum incorporation and achieved a stable, record-breaking performance.^12^

According to the aforementioned achievements, well-aligned one-dimensional (1-D) nanostructures can offer short diffusion distances for minority carrier transport, large surface area for interfacial charge collection, as well as a long optical path for light harvesting. Meanwhile, reactive ballistic deposition (RBD) technique is a versatile method to fabricate highly-oriented and well-defined 1-D nanostructures without introducing undesired impurities. The morphology, surface area and well-aligned nanobundle structure of hematite films can be tailored by the RBD technique.

Introduction of metallic^13^ or nonmetallic^10^ dopants is a promising strategy to increase the electron mobility of hematite for improved photocatalytic efficiency. P stands out as a promising candidate for hematite doping, which substitutes Fe atoms as an n-type dopant. As an electron donor, nonmetallic P possesses more valence electrons than Si, Ti, Co and many other doping elements. Additionally, P–O bonds in hematite exhibit a more covalent nature than other dopant-O bonds, which can effectively avoid the formation of deep electron trapping sites in hematite.^10^ Although P incorporation in hematite has been well recognized to improve the conductivity for better charge transportation, the charge recombination in hematite remains a problem. Thus, an element incorporation strategy that improves the charge separation efficiency in bulk hematite is urgently needed.

This paper describes the design and fabrication of highly oriented hematite nanobundle array with gradient P incorporation using RBD followed by a facile dipping and annealing method. The gradient distributed P concentration in the hematite can increase its conductivity, as well as induce a more upward band bending within a widened region, facilitating charge separation across the radial direction of hematite nanobundle. Further introducing a thin layer of cobalt phosphate (Co-Pi) as cocatalyst can improve the PEC performance of hematite by accelerating the oxygen evolution reaction (OER) kinetics.

## Results and discussion

**Scheme 1:**
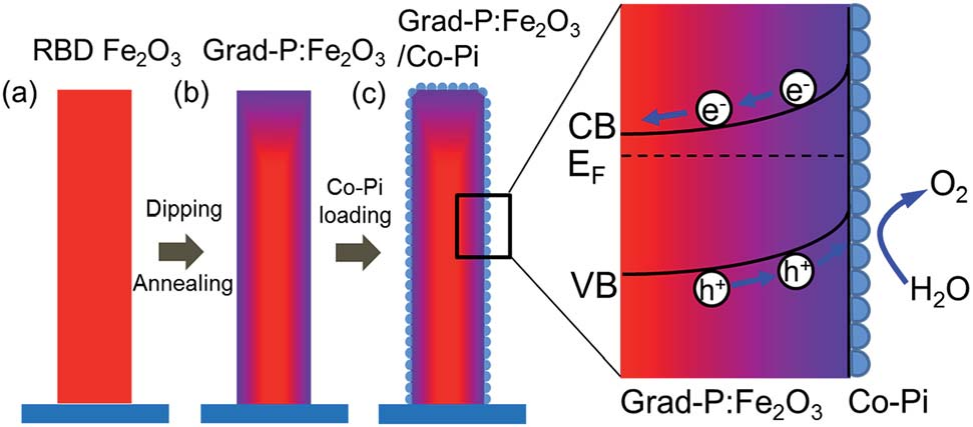
Schematic illustration of the synthetic procedure of a grad-P:Fe_2_O_3_/Co-Pi photoanode. (a) Oriented Fe_2_O_3_ nanobundle arrays grown on FTO substrate using RBD; (b) the prepared Fe_2_O_3_ nanobundle arrays were incorporated with gradient P concentrations (the color gradient as an indicator of P content); (c) a thin Co-Pi layer was loaded on grad-P:Fe_2_O_3_ photoanode.

**Fig. 1:**
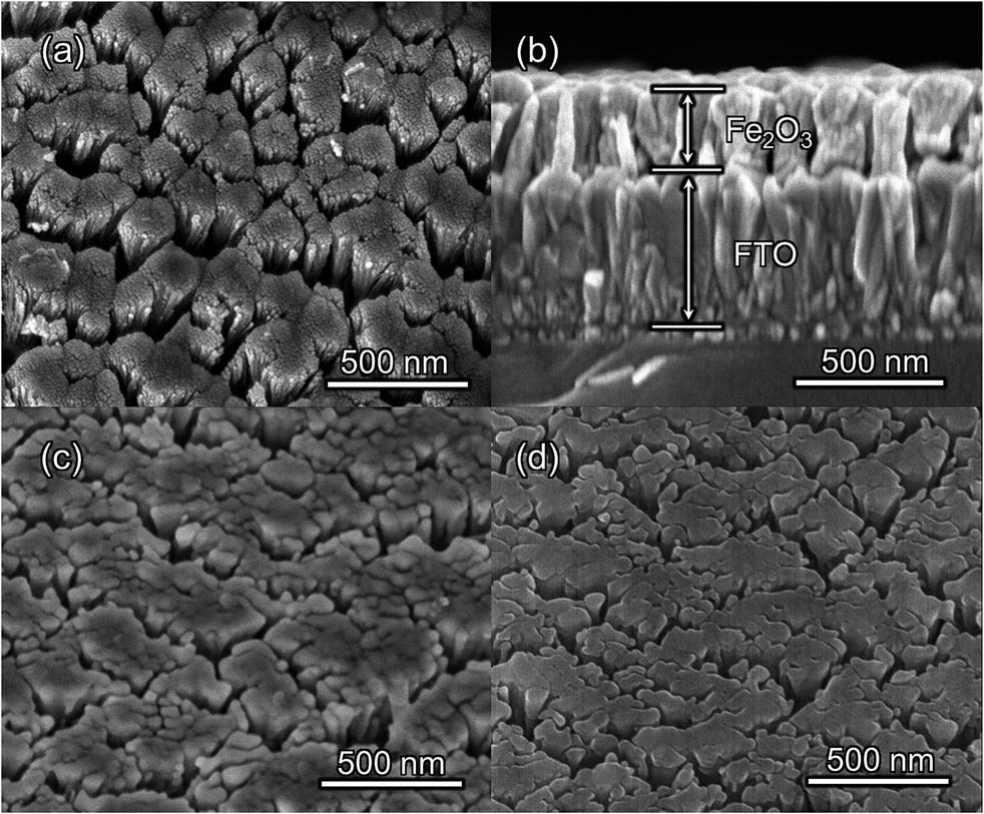
SEM images of (a) top-view and (b) side-view of bare Fe_2_O_3_ (500 °C for 2 h); top-view of (c) bare Fe_2_O_3_ (750 °C for 10 min after 500 °C for 2 h) and (d) grad-P:Fe_2_O_3_ (750 °C for 10 min after 500 °C for 2 h).

The fabrication process of the Fe_2_O_3_ photoanode is illustrated in Scheme 1. Highly oriented Fe_2_O_3_ nanobundle arrays (Fig. 1a) were grown on fluorine-doped tin oxide (FTO) glass substrate employing a homemade RBD system by evaporating metallic Fe metal in a high vacuum chamber with oxygen as the reactive gas (Scheme 1a). The prepared 1-D Fe_2_O_3_ nanobundle arrays are expected to be efficient in charge transportation by shortening the pathway the minority carriers have to travel. The minority carriers could flow through a direct radial pathway of the nanobundle. The P precursor was then introduced to establish a gradient incorporation concentration in Fe_2_O_3_ nanobundles (denoted as grad-P:Fe_2_O_3_), which was achieved *via* a facile solution immersion combined with a short-time thermal treatment (750 °C for 10 min, details in the Experimental section) (Scheme 1b). Subsequently, a thin layer of Co-Pi cocatalyst was deposited to accelerate the water-oxidation reaction on the surface of Fe_2_O_3_ (grad-P:Fe_2_O_3_/Co-Pi) (Scheme 1c). The use of element doping/incorporation to enhance the conductivity is a well-established concept in semiconductor physics.^14^ Although the promoted charge conductivity of Fe_2_O_3_ has been well recognized by element doping/incorporation, the high charge recombination rate in bulk Fe_2_O_3_ still remains a challenge. Hence we propose to carefully control the P doping concentration with a gradient profile in Fe_2_O_3_ to widen the band bending region, thus promoting the charge separation efficiency in the bulk.

In our study, short-time thermal treatment (750 °C for 10 min) can induce gradient P incorporation, where the P concentration decreases gradually from the surface to the core of the Fe_2_O_3_ nanobundles. As will be discussed later, the gradient change of P concentration in the Fe_2_O_3_ nanobundle can widen the band bending region, which improves the separation of charge carriers, for enhanced PEC performance.^15^ On the other hand, a relatively long-time thermal treatment (750 °C for 30 min, details in the Experimental section) attains a homogeneous P-incorporated Fe_2_O_3_ photoanode (denoted as homo-P:Fe_2_O_3_).^16^

**Fig. 2:**
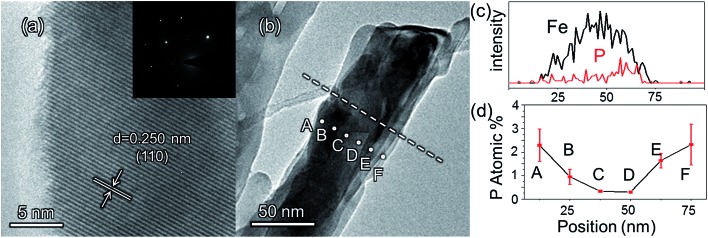
(a) HRTEM image of a selected area of grad-P:Fe_2_O_3_; inset of (a) is the SAED pattern; (b) TEM image of a single grad-P:Fe_2_O_3_ bundle; (c) the P element distribution in a Fe_2_O_3_ nanobundle (line in image b); (d) the P element concentration along the radial direction (dots in image b) (the error bar represents the standard deviation).

The morphologies of the as-prepared bare Fe_2_O_3_ and grad-P:Fe_2_O_3_ nanobundle array thin films were examined by scanning electron microscopy (SEM) (Fig. 1). Tilted Fe_2_O_3_ nanobundle arrays are found to be well-aligned on the FTO substrate (Fig. 1a). The thickness of these thin films is 200 ± 10 nm according to the side-view SEM (Fig. 1b). Comparing the morphology of the Fe_2_O_3_ thin films with and without P incorporation, slight aggregation and shrinkage occur upon the thermal treatment (750 °C for 10 min) (Fig. 1c and d). This rapid thermal treatment induces gradient concentration of P in Fe_2_O_3_ as well as increasing the crystallinity of Fe_2_O_3_. The improved crystallinity of Fe_2_O_3_ upon annealing at 750 °C has been confirmed by high-resolution transmission electron microscopy (HRTEM) (ESI, Fig. S1†). The visual color of both bare Fe_2_O_3_ and grad-P:Fe_2_O_3_ nanobundle array thin films are reddish (ESI, Fig. S2†), and there is no remarkable color change on Fe_2_O_3_ thin films upon P incorporation. These thin films also show similar crystal structure (ESI, Fig. S3a†) and optical properties (ESI, Fig. S3b†). The absorption edges are at about 610 nm, corresponding to the bandgap energy of Fe_2_O_3_. Therefore, morphology and absorption property changes of the grad-P:Fe_2_O_3_ photoanode can be ruled out for improved PEC performance. The TEM images reveal that no apparent lattice distortion of grad-P:Fe_2_O_3_ is observed compared with that of bare Fe_2_O_3_ (ESI, Fig. S4†). The well-aligned diffraction patterns recorded by the corresponding selected area electron diffraction (SAED) (inset, Fig. 2a) indicate the high crystallinity of grad-P:Fe_2_O_3_. The HRTEM image also shows a fine crystalline character without any grain boundaries (Fig. 2a). In addition, the Fe_2_O_3_ displays a lattice spacing of 0.250 nm, corresponding to the (110) plane. This observation confirms that thermal treatment is of great importance to improve the crystallinity of Fe_2_O_3_.

In order to confirm that P element has been incorporated in hematite, Raman analysis was performed. The Raman peaks around 660 cm^–1^ in homo- and grad-P:Fe_2_O_3_ could be ascribed to the disorder phase induced by P incorporation,^17^ whereas this peak is absent in bare Fe_2_O_3_ (ESI, Fig. S5†). A complete elemental energy-dispersive X-ray spectroscopy (EDX) mapping over a grad-P:Fe_2_O_3_ nanobundle was conducted (ESI, Fig. S6b–d†) to illustrate that the Fe_2_O_3_ nanobundle can be fully incorporated by P dopant from the top to the bottom. Both Raman and overview EDX mapping indicate the presence of P element in hematite.

**Fig. 3:**
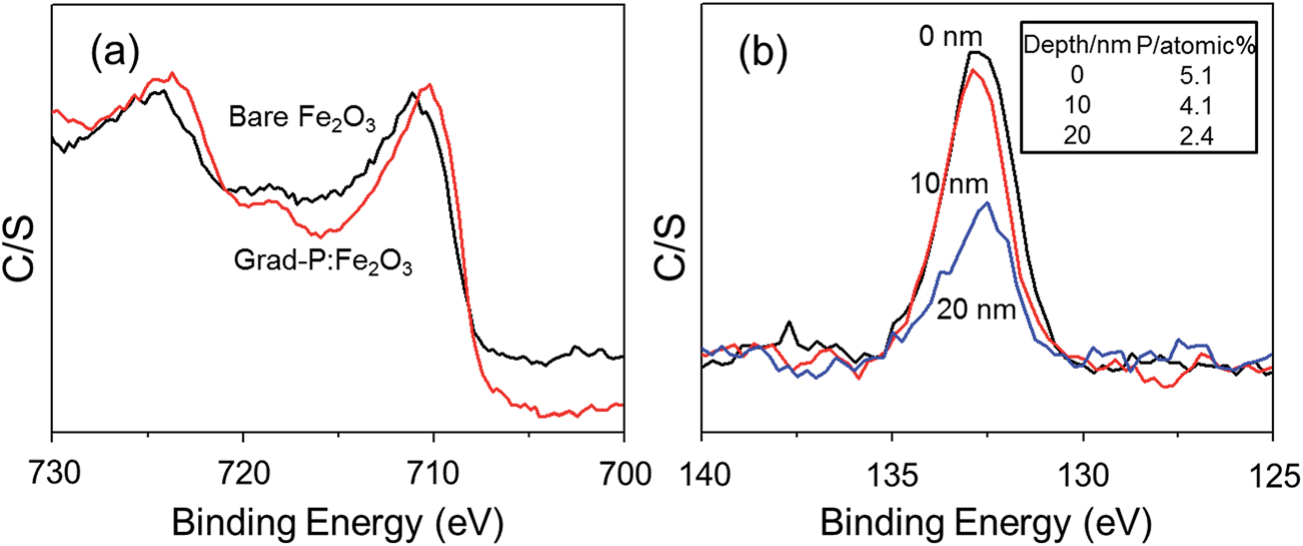
(a) XPS spectra of the Fe 2p signal in bare Fe_2_O_3_ and grad-P:Fe_2_O_3_ photoanodes; (b) XPS depth profile signal of P 2p in grad-P:Fe_2_O_3_, where the P concentration decreases with increasing sputtering time (inset shows the P atomic concentration values at different depth).

The detailed P element distribution in the Fe_2_O_3_ nanobundle (Fig. 2b) was examined by the compositional line-profile scan of EDX (Fig. 2c). It is obvious that the P concentration exhibits a gradient decline from the surface towards the core of Fe_2_O_3_ nanobundle along the radial direction. A series of spots across the bundle by EDX analysis also reveal the concentration profile of P across the Fe_2_O_3_ nanobundle along the radial direction (Fig. 2d). To further verify the P element distribution in the grad-P:Fe_2_O_3_, X-ray photoelectron spectroscopy (XPS) depth analysis was performed by employing argon-ion sputtering. Before argon-ion sputtering, the atomic concentration of P was about 5.1% at the surface of the as prepared grad-P:Fe_2_O_3_. Upon the argon-ion sputtering of 2 and 4 minutes (the sputtering depths are approximately 10 and 20 nm, details in Experimental section), the atomic concentration of P decreased to 4.1 and 2.4%, respectively (inset, Fig. 3b). Based on the above analysis, it is clear that the P concentration presents a gradient profile in the grad-P:Fe_2_O_3_ sample.

XPS also reveals the chemical states of the grad-P:Fe_2_O_3_. Upon P doping, the Fe 2p binding energy shifts to 709.78 eV (Fig. 3a), while P 2p exhibits a binding energy of 132.68 eV (Fig. 3b). The Fe 2p and P 2p signals of grad-P:Fe_2_O_3_ are close to that of FePO_4_,^18^ which indicates a strong interaction between the Fe and P in P:Fe_2_O_3_. According to the crystal structure (ESI, Fig. S3a†) and optical properties (ESI, Fig. S3b†), as well as the chemical states of the grad-P:Fe_2_O_3_ compared with FePO_4_, it is clear that P was well incorporated in the grad-P:Fe_2_O_3_ thin film.

**Fig. 4:**
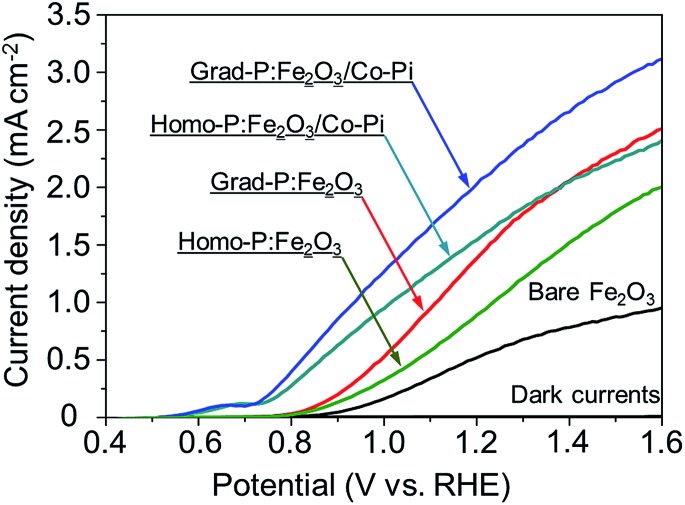
*I*–*V* characteristics of bare Fe_2_O_3_, homo-P:Fe_2_O_3_, grad-P:Fe_2_O_3_, homo-P:Fe_2_O_3_/Co-Pi and grad-P:Fe_2_O_3_/Co-Pi photoanodes measured in 1 M KOH solution under AM 1.5G illumination (100 mW cm^–2^). The dark currents are negligible for all samples.

PEC water oxidation performance of bare Fe_2_O_3_, homo-P:Fe_2_O_3_ and grad-P:Fe_2_O_3_ photoanodes were evaluated by measuring photocurrent density–potential (*I*–*V*) characteristics using a standard three-electrode configuration under air mass (AM) 1.5G illumination (100 mW cm^–2^) (Fig. 4). The bare Fe_2_O_3_ photoanode was prepared *via* identical treatment conditions as that of grad-P:Fe_2_O_3_ photoanode without P incorporation. The photocurrent density of the bare Fe_2_O_3_ photoanode is 0.58 mA cm^–2^ at 1.23 V (*vs.* RHE), which is comparable to reported values in the literature.^19^

It is found that the photocurrent density of P-incorporated Fe_2_O_3_ nanobundle arrays is closely related to the P content (ESI, Fig. S7†; P content in our study was determined by the EDX analysis). The photocurrent density of homo-P:Fe_2_O_3_ nanobundle arrays is enhanced as the P atomic concentration is increased from 0 to 2%, and then decreases with further increase of P atomic ratio. The homo-P:Fe_2_O_3_ (2%) exhibits a photocurrent density of 1.10 mA cm^–2^ at 1.23 V (*vs.* RHE), which is doubled compared to the bare Fe_2_O_3_ nanobundle. It is noteworthy that the grad-P:Fe_2_O_3_ photoanode shows a higher photocurrent density of 1.48 mA cm^–2^ at 1.23 V (*vs.* RHE), which is superior to that of the homo-P:Fe_2_O_3_ photoanode. In order to rule out that the lower PEC performance of homo-P:Fe_2_O_3_ is due to the damage of the conductive FTO layer upon longer thermal treatment (30 min *vs.* 10 min), we have carefully examined the resistivity of the FTO glass upon various annealing conditions using a four-point probe. The resistivity for all these FTO samples are on the same order of 10^–4^ Ω cm (ESI, Table S1†), indicating that the conductivity of FTO could be well preserved in the thermal treatment in this study. Specifically, the resistivity of FTO which is subjected to the annealing condition for grad- and homo-P:Fe_2_O_3_, is very similar (∼8.7 and 9.2 × 10^–4^ Ω cm, respectively), suggesting the lower PEC performance of homo-P:Fe_2_O_3_ is not due to the FTO substrate. The *I*–*V* characteristics for these photoanodes were measured with 10 repeated samples to demonstrate the sample-to-sample variation. The photocurrent values at 1.23 V (*vs.* RHE) were compared, with standard deviations marked as error bars, to verify the reliability of photocurrent improvement upon P-doping (ESI, Fig. S8†).

**Fig. 5:**
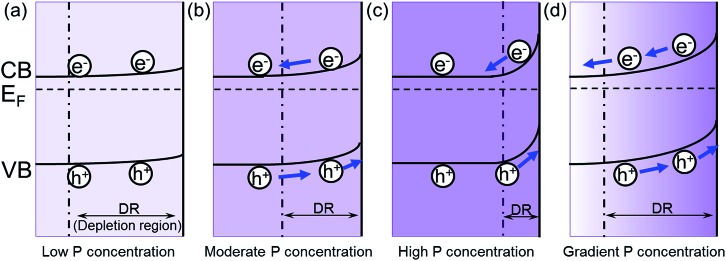
The effect of P concentration on the band bending in hematite. The band bending of (a) low P concentration; (b) moderate P concentration; (c) high P concentration; (d) gradient P concentration. *E*_F_ is the Fermi level relative to the estimated conduction band (CB) and valence band (CB) edges as previously reported,^22,23^ and the boundary for the depletion region is marked by the dashed line.

The increase of carrier density can increase the band bending at the semiconductor/electrolyte interface, which could facilitate the charge transfer.^20^ When the P content is low, the band bending is small. Therefore, the excited electron/hole cannot be separated effectively in the Fe_2_O_3_ (Fig. 5a). As the P content is increased, the band bending will also increase, which improves the separation of electron/hole pairs (Fig. 5b). Meanwhile, the width of depletion layer will decrease with increasing P content. Thus, with too much P content, the depletion region will become too narrow for effective charge separation (Fig. 5c). In addition, the high concentration of dopant may lead to the segregation of dopant phase, which will serve as recombination centers trapping carriers.^21^ Consequently, further increase of P content will hinder the photocatalytic efficiency. Therefore, the photocurrent will increase with the increased P content in the hematite until it reaches a maximum value with a moderate P content in hematite, and then the photocurrent will begin to decline due to the side effects with excessive P content.

In the grad-P:Fe_2_O_3_ nanobundle, the P concentration exhibits a gradient decline from the surface towards the core of Fe_2_O_3_ nanobundle along the radial direction. As illustrated in the band bending schematic (Fig. 5c), the gradient profile of P content will lead to increasing band bending from the Fe_2_O_3_ core towards the surface, hence widening the total band bending region. Therefore, a more upward band bending from the bulk to the surface over a large region is formed.^20,22^ The degree of band banding at the surface region of grad-P:Fe_2_O_3_ is more significant compared with moderately doped homo-P:Fe_2_O_3_ (Fig. 5d *vs.* b), and comparable with heavily doped homo-P:Fe_2_O_3_ (Fig. 5d *vs.* c). Meanwhile, the width of band bending region of the grad-P:Fe_2_O_3_ is prolonged compared with heavily doped homo-P:Fe_2_O_3_ (Fig. 5d *vs.* c). Previous studies have revealed that the electric field in the band bending region of gradient doped GaN would be remarkably enhanced,^22^ and the hole diffusion length is also increased in the electric field induced by GaAs with gradient doping.^23^ Thus, charge separation is expected to be greatly enhanced in the grad-P:Fe_2_O_3_ electrode with a gradient P concentration.

Based on the analysis above, it is speculated that the incorporation of P plays two important roles in this work. On the one hand, P doping substantially increases the electron density of Fe_2_O_3_ and greatly boosts its conductivity, which is the case for both homo- and grad-P:Fe_2_O_3_ samples. On the other hand, the gradient distribution of P induces a more upward band bending within a widened region, improving the charge separation efficiency in bulk Fe_2_O_3_. Therefore, the PEC performance of grad-P:Fe_2_O_3_ exceeds that of homo-P:Fe_2_O_3_ with an additional charge separation effect in bulk Fe_2_O_3_.

**Fig. 6:**
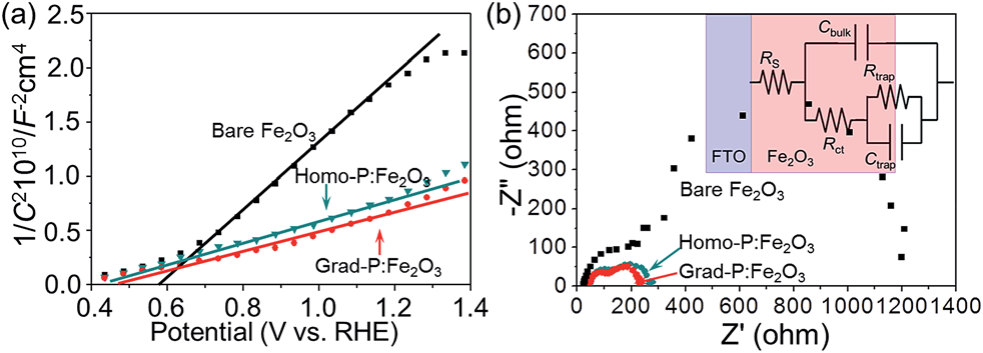
(a) Mott–Schottky plots and (b) EIS Nyquist plots of bare Fe_2_O_3_, homo-P:Fe_2_O_3_ and grad-P:Fe_2_O_3_ photoanodes (inset: equivalent circuit of the photoanode).

In order to clarify the enhanced charge transportation upon P incorporation in homo- and grad-P:Fe_2_O_3_, Mott–Schottky analysis was conducted. The slopes of the Mott–Schottky plot of P:Fe_2_O_3_ photoanodes are substantially smaller than that of bare Fe_2_O_3_ (Fig. 6a), and the positive slopes suggest that P atoms act as n-type dopants in hematite. Although the Mott–Schottky analysis is derived from a planar electrode model, it is still reasonable to calculate the carrier density for comparison purposes.^24^ The carrier densities calculated from the slopes of the Mott–Schottky plots for bare Fe_2_O_3_, homo- and grad-P:Fe_2_O_3_ photoanodes are 1.9 × 10^20^ cm^–3^, 5.4 × 10^20^ cm^–3^ and 5.8 × 10^20^ cm^–3^, respectively. Although a more noticeable increase of carrier density is theoretically expected for a P atomic concentration larger than 1%, the three-fold increase in this study is likely due to the non-single crystalline nature of Fe_2_O_3_, as well as the incomplete activation of incorporated P dopants. Nevertheless, the higher and comparable carrier densities of homo- and grad-P:Fe_2_O_3_ compared with bare Fe_2_O_3_ clearly indicate that P incorporation is effective to improve the electrical conductivity of Fe_2_O_3_ and the charge transportation is similar in grad-P:Fe_2_O_3_ and homo-P:Fe_2_O_3_.

To further verify the similar charge transport property between grad-P:Fe_2_O_3_ and homo-P:Fe_2_O_3_, Nyquist plots of electrochemical impedance spectroscopy (EIS) were attained under AM 1.5G illumination (100 mW cm^–2^), with a bias of 1.23 V (*vs.* RHE) (Fig. 6b). An equivalent circuit model was built to fit the EIS data (inset, Fig. 6b), where *R*_s_ represents all the series resistances in the electrochemical cell, *R*_ct_ represents the resistance in the Fe_2_O_3_, *R*_trap_ represents the charge resistance across the semiconductor/electrolyte interface, *C*_bulk_ represents the capacitance in the depletion layer of the semiconductor, and *C*_trap_ represents the capacitance at the surface of the semiconductor. In this case, a smaller *R*_ct_ indicates a lower charge-transfer resistance in the Fe_2_O_3_. The fitting results show that both grad-P:Fe_2_O_3_ (105.3 ± 2.3 Ω) and homo-P:Fe_2_O_3_ (111.2 ± 2.2 Ω) show comparable improved conductivity compared with bare Fe_2_O_3_ (213.1 ± 7.3 Ω) owning to the P incorporation (ESI, Table S2†). In another attempt to verify the effectiveness of resistivity reduction upon P incorporation, electrode assemblies (FTO/Fe_2_O_3_/Al, details in Experimental section) with planar homo- and grad-P:Fe_2_O_3_ films were fabricated for solid-state *I*–*V* characterization (ESI, Fig. S9†). The similar slope increase in solid-state *I*–*V* curves for homo- and grad-P:Fe_2_O_3_ indicate a significant conductivity improvement compared with bare Fe_2_O_3_, and our homogenous and gradient doping approach will not result in noticeable conductivity difference. Based on the Mott–Schottky and EIS analysis, as well as the solid-state *I*–*V* characterization, it can be confirmed that P doping equally boosts the conductivity of grad- and homo-P:Fe_2_O_3_.

**Fig. 7:**
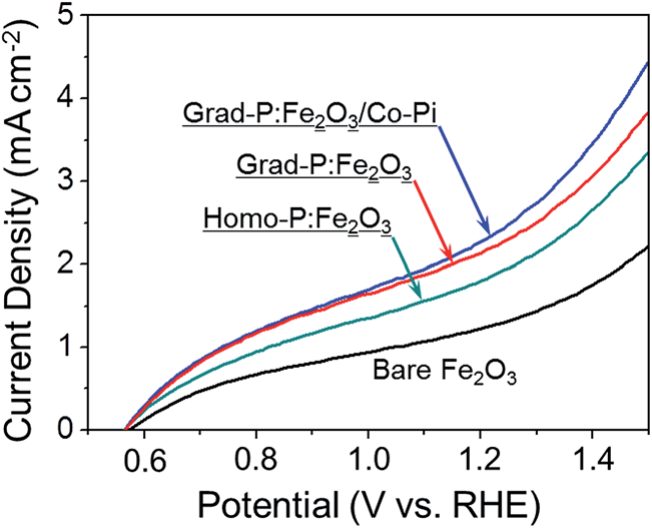
*I*–*V* characteristics of bare Fe_2_O_3_, homo-P:Fe_2_O_3_, grad-P:Fe_2_O_3_ and grad-P:Fe_2_O_3_/Co-Pi photoanodes measured for H_2_O_2_ oxidation under AM 1.5G illumination. The dark currents were negligible for all samples.

To confirm the existence of an additional charge separation effect caused by gradient P incorporation, as indicated by the band diagram analysis (Fig. 5), 0.5 M H_2_O_2_ was added into the electrolyte as a hole scavenger for *I*–*V* measurement under AM 1.5G (Fig. 7).^25^ The reaction kinetics in the presence of H_2_O_2_ hole scavenger is equally fast for all samples. In this circumstance, the photocurrent of grad-P:Fe_2_O_3_ is remarkable higher than that of homo-P:Fe_2_O_3_, even though these two samples exhibit similar conductivity. This result suggests that the improved photocurrent is very likely attributed to an extra charge separation effect caused by the gradient P incorporation in Fe_2_O_3_.

**Fig. 8:**
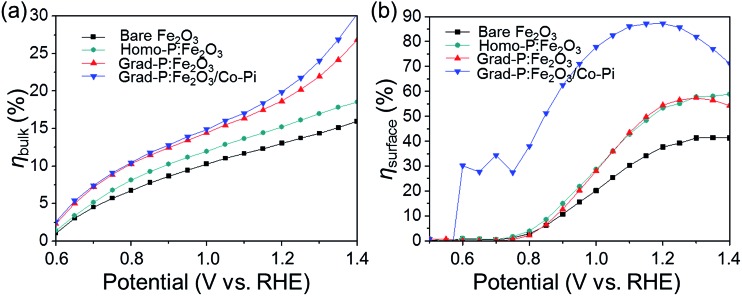
Charge separation efficiency (a) in the bulk (*η*_bulk_) and (b) on the surface (*η*_surface_) of photoanodes.

To further confirm the additional charge separation effect caused by gradient P incorporation, the efficiency of charge transport in the bulk (*η*_bulk_, relating to bulk charge separation) was calculated (Fig. 8a, details in Experimental section). The *η*_bulk_ of bare Fe_2_O_3_ photoanode is ∼11% at 1.23 V (*vs.* RHE), which is comparable to the previously reported value (∼13%).^26^ Upon P doping, the *η*_bulk_ of grad-P:Fe_2_O_3_ reaches ∼22% at 1.23 V (*vs.* RHE), which is doubled compared to that of bare Fe_2_O_3_. The *η*_bulk_ of grad-P:Fe_2_O_3_ is also higher than that of homo-P:Fe_2_O_3_ (∼16%) at 1.23 V *vs.* RHE, which could be attributed to the improved charge separation efficiency in the bulk of grad-P:Fe_2_O_3_. Considering the similar conductivity between grad-P:Fe_2_O_3_ and homo-P:Fe_2_O_3_, it could be inferred that gradient P incorporation results in an extra promotive effect for Fe_2_O_3_ that facilitates charge separation.

To further improve the PEC performance of the grad-P:Fe_2_O_3_ photoanode, we adopted Co-Pi as an OER cocatalyst to accelerate water oxidation on the surface.^27^ The Co-Pi cocatalyst thin layer is nearly optically transparent on the Fe_2_O_3_, which is confirmed by the optical image (ESI, Fig. S2†) and UV-vis spectra (ESI, Fig. S3b†). As a result, the grad-P:Fe_2_O_3_/Co-Pi photoanode could reach a photocurrent density of 2.0 mA cm^–2^ at 1.23 V (*vs.* RHE). A remarkable cathodic shift in the onset potential from 0.85 V (*vs.* RHE) for bare Fe_2_O_3_ and grad-P:Fe_2_O_3_ photoanodes to 0.58 V (*vs.* RHE) for this grad-P:Fe_2_O_3_/Co-Pi photoanode is attained.

To quantify the contributions of gradient P incorporation and the OER cocatalyst (Co-Pi) to the promotion of surface reaction kinetics, the efficiency of surface charge transfer (*η*_surface_, relating to surface charge separation, *i.e.*, surface reaction kinetics) was calculated as well (Fig. 8b, details in Experimental section). The *η*_surface_ for grad-P:Fe_2_O_3_ (∼57% at 1.23 V *vs.* RHE) and homo-P:Fe_2_O_3_ (∼57% at 1.23 V *vs.* RHE) are almost identical, which is higher than that of bare Fe_2_O_3_ (∼40% at 1.23 V *vs.* RHE), illustrating that the severe surface recombination and sluggish surface reaction for Fe_2_O_3_ are partially alleviated upon P incorporation. It is worth noting that the similar *η*_surface_ for grad- and homo-P:Fe_2_O_3_ indicates that their surface activity is almost the same. This result is consistent with the phenomenon that the onset potential of homo-P:Fe_2_O_3_ is identical to that of grad-P:Fe_2_O_3_ (Fig. 4). Upon the loading Co-Pi cocatalyst, *η*_surface_ of the grad-P:Fe_2_O_3_/Co-Pi sample is greatly enhanced in the whole potential range (0.6–1.4 V *vs.* RHE) and achieves ∼88% at 1.23 V (*vs.* RHE). This significant *η*_surface_ improvement indicates the reduced surface recombination losses upon Co-Pi loading, which may result either from faster water oxidation or slower recombination.

**Fig. 9:**
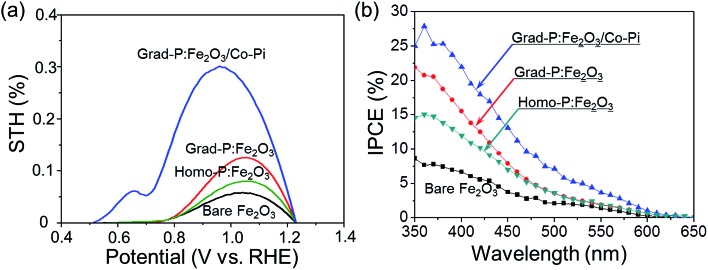
(a) Photoconversion efficiency as a function of applied potential; (b) IPCE in the region 350–650 nm at a bias of 1.23 V (*vs.* RHE) for bare Fe_2_O_3_, homo-P:Fe_2_O_3_, grad-P:Fe_2_O_3_ and grad-P:Fe_2_O_3_/Co-Pi photoanodes.

To quantitatively evaluate the efficiency of PEC water splitting of these photoanodes, the photoconversion efficiency is calculated (Fig. 9a). A maximum photoconversion efficiency of 0.32% is observed for the grad-P:Fe_2_O_3_/Co-Pi photoanode at 0.92 V (*vs.* RHE) while the efficiencies for bare Fe_2_O_3_, homo- and grad-P:Fe_2_O_3_ photoanodes are 0.05, 0.09 and 0.12% at 1.05 V (*vs.* RHE), respectively. Furthermore, incident photon-to-electron conversion efficiency (IPCE) was also obtained to assess the contribution of monochromatic light to the current density at wavelengths ranging from 350 to 650 nm at a potential of 1.23 V (*vs.* RHE) in 1 M KOH solution (Fig. 9b). The grad-P:Fe_2_O_3_ photoanode exhibits significantly enhanced IPCE in comparison with bare Fe_2_O_3_ photoanode in the absorption region of Fe_2_O_3_, suggesting that the absorbed photons can be utilized more efficiently, due to the improved electrical conductivity and charge separation of the grad-P:Fe_2_O_3_.

**Fig. 10:**
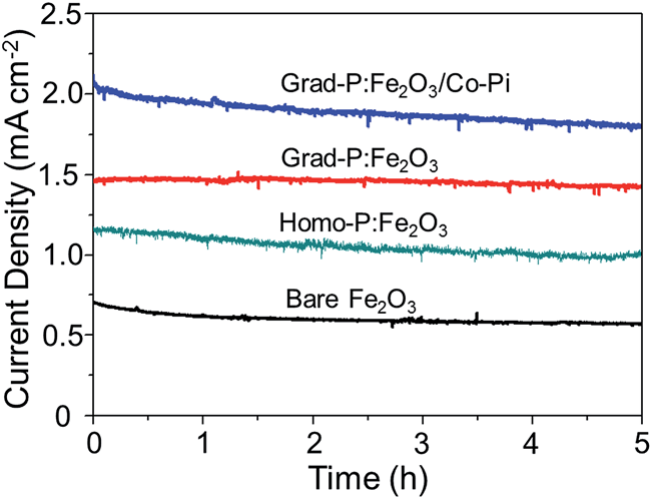
Stability test of bare Fe_2_O_3_, homo-P:Fe_2_O_3_, grad-P:Fe_2_O_3_ and grad-P:Fe_2_O_3_/Co-Pi photoanodes for 5 h at 1.23 V (*vs.* RHE) under AM 1.5G illumination.

A 5 h stability test of these photoanodes was performed at 1.23 V (*vs.* RHE) under AM 1.5G illumination (Fig. 10). The result shows that all these photoanodes retained acceptable stability during 5 h. In the given 5 h experimental time duration, the photocurrent density of the bare Fe_2_O_3_ photoanode shows a slight decrease from 0.58 to 0.53 mA cm^–2^. However, the grad-P:Fe_2_O_3_ photoanode shows an impressive stability during the testing time, keeping a steady photocurrent density of 1.48 mA cm^–2^ (the steady photocurrent density of the homo-P:Fe_2_O_3_ photoanode is 1.10 mA cm^–2^). The photocurrent density of the grad-P:Fe_2_O_3_/Co-Pi photoanode shows an apparent decrease from 2.0 to 1.78 mA cm^–2^. The decline of this grad-P:Fe_2_O_3_/Co-Pi photoanode is most likely due to the decomposition of the Co-Pi water-oxidation cocatalyst.

## Conclusions

We have demonstrated the design and fabrication of effective, stable and well-defined 1-D hematite nanoarray thin films with gradient P incorporation in the radial direction, using a RBD technique followed by a facile dipping and annealing method. An AM 1.5G photocurrent of 2.0 mA cm^–2^ at 1.23 V (*vs.* RHE) for water oxidation was achieved. EDX quantitative analysis and XPS depth profile verify that the P element indeed has a gradient concentration profile in the radial direction. The Mott–Schottky, EIS analysis and the solid-state *I*–*V* characterization, as well as the *I*–*V* characteristics with H_2_O_2_ as the hole scavenger clearly show that the improved PEC performance can be ascribed to the enhanced charge separation of the gradient P incorporation in the Fe_2_O_3_, which increases the band bending over a large region. Higher water-oxidation efficiency and lower onset potential can be achieved by loading appropriately designed Co-Pi OER cocatalysts. We believe that this gradient doping strategy can be applied to other semiconductor photoelectrodes for improved performance of solar water splitting.

## Experimental section

### Reactive ballistic deposition of porous hematite films

A homemade RBD system was used to prepare the porous hematite thin films. The base pressure of the high vacuum chamber of RBD system is around 5 × 10^–8^ mbar. Oxygen as reactive gas was introduced to backfill the vacuum chamber to about 1 × 10^–6^ mbar using a leak valve. A homemade e-beam evaporator was used to evaporate an iron rod (minimum purity of 99.95%) with the iron rod mounted on the top of the e-beam evaporator directly near the tungsten filament. The Fe evaporation was conducted in the O_2_ ambient at about 1 × 10^–6^ mbar. FTO coated glasses were used as the substrates. The sample holder could rotate by a full 360 degrees, making it possible to have arbitrary deposition angles. The distance between the evaporation source and the substrate was approximately 4 inches. A quartz crystal microbalance was used to monitor the deposition rates to estimate the thickness of the deposited films. In our preparation, we fixed a deposition rate of about 2 nm min^–1^, and the deposition time was set at 100 min to attain a thickness of 200 nm.

The gradient P-incorporated porous Fe_2_O_3_ thin films were prepared by a dipping and annealing treatment. Before the treatment, the porous Fe_2_O_3_ thin films were annealed in air at 500 °C for 2 h. The rate of heating was 5 °C min^–1^ and then the samples were allowed to cool to room temperature naturally. First, the annealed Fe_2_O_3_ thin films were soaked in 0.2 M Na_3_PO_4_ solution for 10 min, and then the wet films were dried in a drying oven at 100 °C for more than 1 h. After the pre-treatment, these samples were further annealed in 750 °C for 10 min to induce a gradient P incorporation in Fe_2_O_3_. Finally, these samples were washed thoroughly with deionized water to remove any residual impurities.

Co-Pi OER cocatalyst was loaded on the samples by a photo-assisted electrodeposition method. The Fe_2_O_3_ thin film was immersed in 0.5 mM Co(NO_3_)_2_ in 0.1 M K_2_HPO_4_ buffer at pH 7, which was purged for more than 1 h by nitrogen. A 300 W xenon lamp (Beijing Perfectlight Technology Co. Ltd; LS-SXE300CUV) equipped with an AM 1.5G filter was used as the light source to obtain simulated sunlight. The power intensity of the simulated sunlight was calibrated to 100 mW cm^–2^. The photo-assisted electrodeposition was conducted at a bias of 0.4 V *vs.* Ag/AgCl electrode (1.0 V *vs.* RHE) for 2 min.

### TEM sample preparation

The Fe_2_O_3_ nanobundle arrays on the FTO substrate were scraped off carefully in a 20 ml beaker with a sharp blade, and then 1.5 ml isopropanol was added as solvent to obtain a suspension. The suspension was dispersed evenly by using ultrasound for 20 min. The as-prepared suspension was dropped on an ultrathin carbon film coated copper wire mesh for TEM characterization.

### Structural characterization

The morphology and nanostructure of the samples were characterized by field emission scanning electron microscopy (FE-SEM, Hitachi S-4800, 5 kV) and transmission electron microscopy (TEM, JEM 2100 F, 200 kV). The crystal structure of the samples were investigated by an X-ray diffractometer (type D/MAX 2500) equipped with a nickel-filtered Cu-Kα radiation (*λ* = 1.5416 Å) source at 40 kV and 140 mA. The XRD spectra were collected at a scanning speed of 0.02° per step over a 2*θ* range from 20 to 80°. X-Ray photoelectron spectra were recorded on a Physical Electronics PHI 1600 ESCA system with an Al-Kα X-ray source (*E* = 1486.6 eV). Optical reflectance and transmittance properties of the samples were recorded on a Shimadzu UV-2550 spectrophotometer.

XPS depth analysis was performed on a gradient P-incorporated planar Fe_2_O_3_ thin film by the Physical Electronics PHI 1600 ESCA system, and the sputtering depth was calibrated to be 5 nm min^–1^ at a silica wafer as the reference.

### Photoelectrochemical tests

The PEC properties of the samples were evaluated using a three-electrode system. The samples were set as the working electrode; saturated Ag/AgCl and platinum foil (2 cm × 2 cm area) were used as the reference electrode and the counter electrode, respectively. The electrolyte was a 1.0 M KOH aqueous solution. *I*–*V* characteristics of the electrode were performed on an electrochemical workstation (IVIUM CompactStat.e20250) at a scan rate of 50 mV s^–1^. The samples were packaged using black tapes with an exposed area of 1 cm^2^. A 300 W xenon lamp (Beijing Perfectlight Technology Co. Ltd; LS-SXE300CUV) equipped with an AM 1.5G filter was used as the light source. The power intensity of the simulated sunlight was calibrated to 100 mW cm^–2^. Prior to *I*–*V* tests, nitrogen purging for more than 30 min was conducted to remove the dissolved oxygen in the electrolyte.

Additionally, we have acquired the solar to hydrogen (STH) efficiencies to evaluate the photocatalytic performance of the photoelectrodes. The calculating equation is as follows:^28^

1 *η* = *I*(1.23 – *V*)*P*_light_

where *I* is the photocurrent density at the measurement applied bias, *V* is the applied bias (*vs.* RHE), and *P*_light_ is the incident light intensity of 100 mW cm^–2^ (AM 1.5G illumination). The measured potential with respect to Ag/AgCl reference electrodes could be converted to an RHE following:^29^

2 *E*_RHE_ = *E*_Ag/AgCl_ + 0.059pH + *E*0Ag/AgCl

where *E*_RHE_ is the converted potential *vs.* RHE, *E*_Ag/AgCl_ is the applied potential against Ag/AgCl reference electrode, and *E*0Ag/AgCl = 0.197 V at 25 °C.

The photocurrent density arising from PEC performance (*J*_PEC_) can be described as following:

3 *J*_PEC_ = *J*_abs_*η*_bulk_*η*_surface_

where *J*_abs_ is the photocurrent density when completely converting the absorbed photons into current (*i.e.*, absorbed photon-to-current efficiency (APCE) = 100%). Adding 0.5 M H_2_O_2_ as the electrolyte can largely suppress the surface recombination of charge carriers without influencing the charge separation in the electrode bulk (*i.e.*, *η*_surface_ could be regarded as 100%). Therefore, *η*_bulk_ and *η*_surface_ can be determined as following:

4 *η*_bulk_ = *J*_H_2_O_2__/*J*_abs_

5 *η*_surface_ = *J*_H_2_O_/*J*_H_2_O_2__

where *J*_H_2_O_ and *J*_H_2_O_2__ are the photocurrent density for PEC H_2_O oxidation and H_2_O_2_ oxidation, respectively. By estimating the overlapped areas between the UV-vis absorption spectrum and the AM 1.5G solar spectrum, assuming APCE = 100%, the *J*_abs_ of Fe_2_O_3_ was calculated to be 11.4 mA cm^–2^. As the ability of light absorption was approximate the same for all the samples, this value is suitable for bare Fe_2_O_3_, homo-P:Fe_2_O_3_, grad-P:Fe_2_O_3_ and grad-P:Fe_2_O_3_/Co-Pi, respectively. Therefore, the charge separation efficiency in the bulk and on the surface of the samples can be determined independently using eqn (3)–(5), as displayed in Fig. 8.

Solid-state *I*–*V* curves were measured on a potentiostat in the voltage range from –1 to 1 V. The testing electrode assemblies are illustrated in the ESI, Fig. S9.† The gradient P distribution along the depth direction in the planar film is similar to that along the radial direction in the nanobundle. The planar samples (bare Fe_2_O_3_, homo-P:Fe_2_O_3_, grad-P:Fe_2_O_3_) were prepared *via* identical conditions to that of the nanobundle photoanodes, except that the evaporation flux was normal to the sample surface. A 300 nm-thick Al film was deposited by e-beam evaporation on top of these samples as the front contact, and the FTO substrate acted as the back contact. Two Cu wires were attached to the Al film and FTO, respectively, for electrical connection with the potentiostat.

The IPCE was obtained using a quantum efficiency/IPCE system (Zolix Solar Cell Scan, 100) under monochromatic light. The IPCE spectra were measured at a constant potential (1.23 V *vs.* RHE), at wavelengths from 350 to 650 nm.

The IPCE is calculated from the current density recorded at different wavelengths using the following formula:^30^

6 IPCE = 1240*j*/(*P*_light_*λ*)

where *j* is the measured current density (mA cm^–2^), *P*_light_ is the calibrated and monochromated illumination power (mW cm^–2^), and *λ* is the wavelength (nm) of the incident light at the measured photocurrent and illumination intensity.

Mott–Schottky measurements were measured in a 1.0 M KOH aqueous solution at a frequency of 10^3^ Hz and scan rate of 10 mV s^–1^. The potential was measured against an Ag/AgCl reference electrode.

The donor concentration was calculated with the following equation:^30^

7 *N*_d_ = (2/*eεε*_o_)[d(1/*C*^2^)/d*V*^–1^]

where *e* = 1.60 × 10^–19^ C is the electron charge, *ε* = 80 is the dielectric constant of hematite, *ε*_o_ = 8.85 × 10^–14^ F cm^–1^ is the vacuum permittivity, *C* is the capacitance of the space charge region, *V* is the electrode applied potential, and *N*_d_ is the donor concentration.

Impedance measurements were measured in a 1.0 M KOH aqueous solution under simulated sunlight illumination over a frequency range from 10^5^ to 10^–1^ Hz. Data were fitted using Zview software (Scribner Associates).

Stability of PEC performance of bare Fe_2_O_3_, homo-P:Fe_2_O_3_, grad-P:Fe_2_O_3_ and grad-P:Fe_2_O_3_/Co-Pi photoanodes was measured at a constant potential of 1.23 V (*vs.* RHE) under AM 1.5G illumination for 5 h.
