# Supplementary material for: Gradient doping of phosphorus in Fe2O3 nanoarray photoanodes for enhanced charge separation
Source: Chem Sci. 2016 Oct 3;8(1):91–100. doi: 10.1039/c6sc03707k (PMC5304616; doi:10.1039/c6sc03707k)
Supplement: Supplementary file 1 [file SC-008-C6SC03707K-s001.pdf]

Electronic Supplementary Material (ESI) for Chemical Science.
This journal is © The Royal Society of Chemistry 2016

Supporting Information

Gradient Doping of Phosphorus in Fe_2O_3 Nanoarray Photoanodes for Enhanced Charge Separation

Zhibin Luo, Chengcheng Li, Shanshan Liu, Tuo Wang* and Jinlong Gong*

Key Laboratory for Green Chemical Technology of Ministry of Education, School of Chemical Engineering and Technology, Tianjin University; Collaborative Innovation Center of Chemical Science and Engineering, Tianjin 300350, China

*Author to whom correspondence should be addressed: jlgong@tju.edu.cn; wangtuo@tju.edu.cn

Fig. S1. HRTEM images of bare Fe_2O_3 (a) annealed at 500 °C for 2 hours, and (b) with further annealed at 750 °C for 10 minutes.

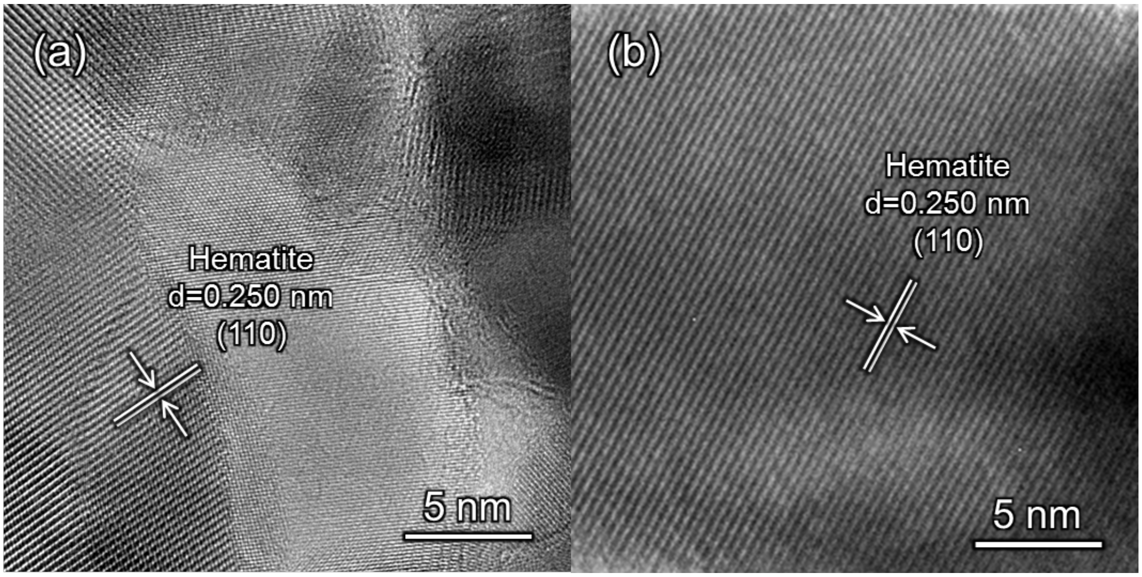

Fig. S2. Digital pictures of (a) bare Fe_2O_3, (b) grad-P:Fe_2O_3 and (c) grad-P:Fe_2O_3/Co-Pi photoanodes.

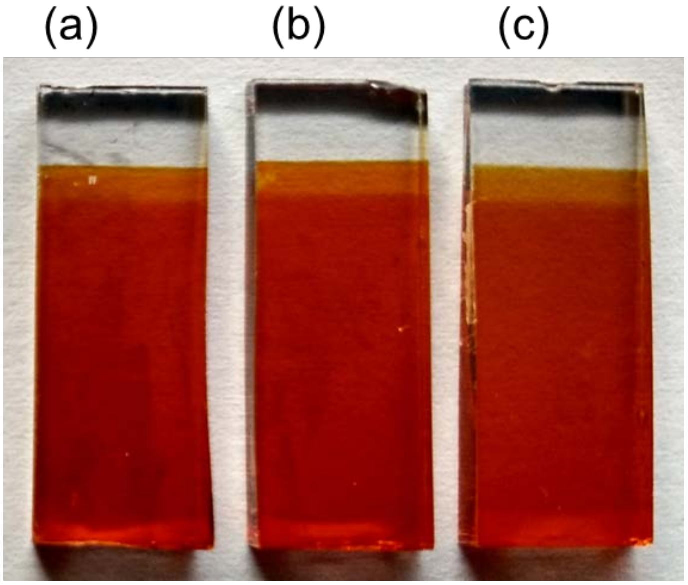

Fig. S3. (a) XRD and (b) UV-vis spectra of bare Fe_2O_3, grad-P:Fe_2O_3 and grad-P:Fe_2O_3/Co-Pi nanobundle array thin films.

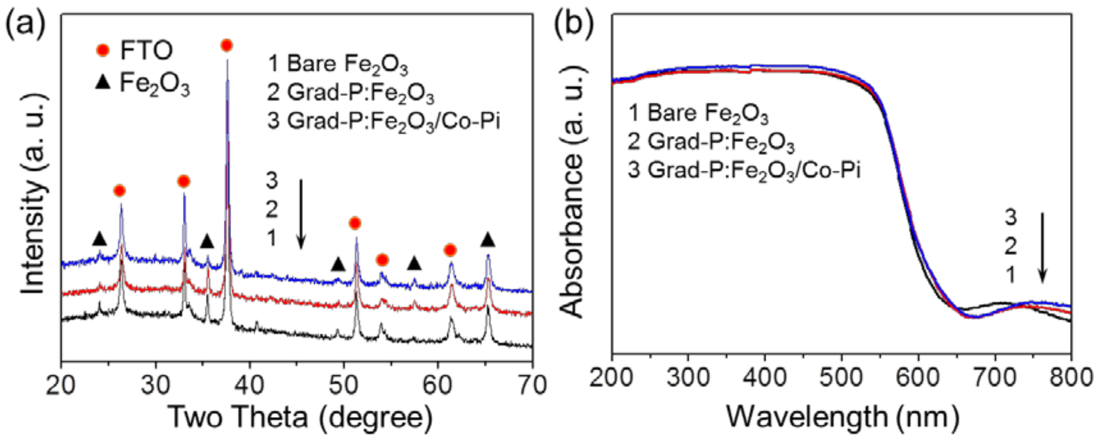

Fig. S4. TEM images of (a) bare Fe_2O_3 and (b) grad-P:Fe_2O_3 photoanodes annealed at 750 °C for 10 minutes.

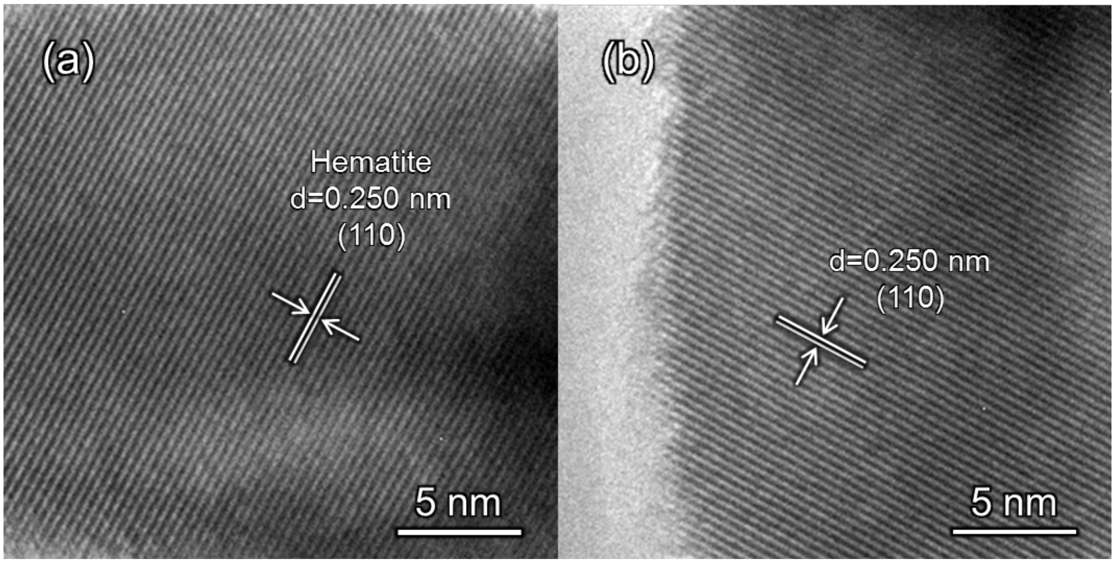

Fig. S5. Raman spectra of bare Fe_2O_3, homo-P:Fe_2O_3 and grad-P:Fe_2O_3 thin films.

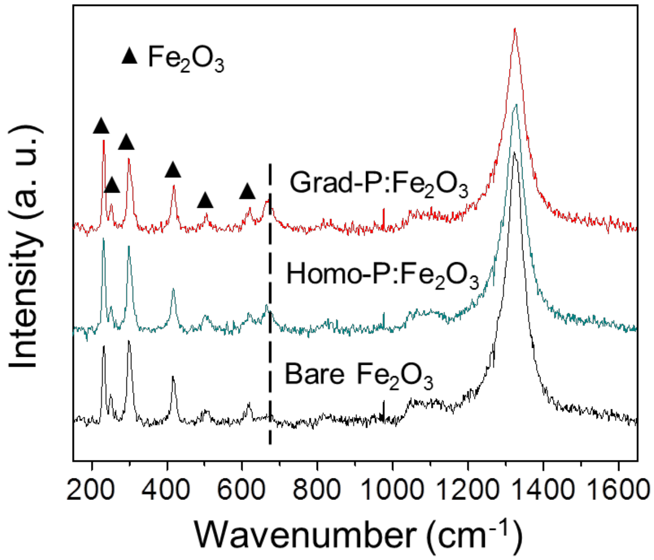

Fig. S6. TEM and EDX characterization of a representative grad-P:Fe_2O_3. (a), selected grad-P:Fe_2O_3 nanobundles. (b), (c), (d), element mapping of P, O and Fe, respectively.

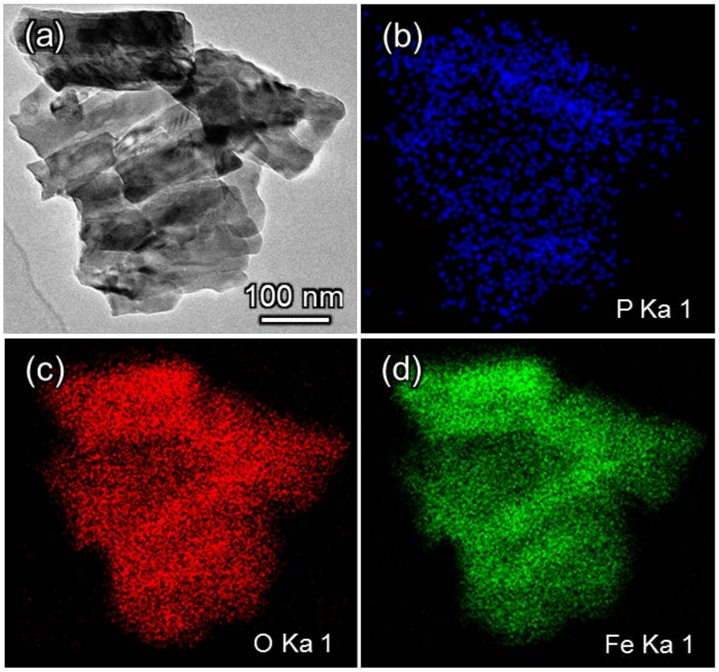

Fig. S7. (a) The P content in homo-P:Fe_2O_3 nanobundle confirmed by EDX analysis. (The error bar indicates the standard deviation) (b) I-V characteristics of P-incorporated Fe_2O_3 photoanodes with different P content. (1: 0.9% homo-P:Fe_2O_3, 2: 2% homo-P:Fe_2O_3, 3: 3% homo-P:Fe_2O_3)

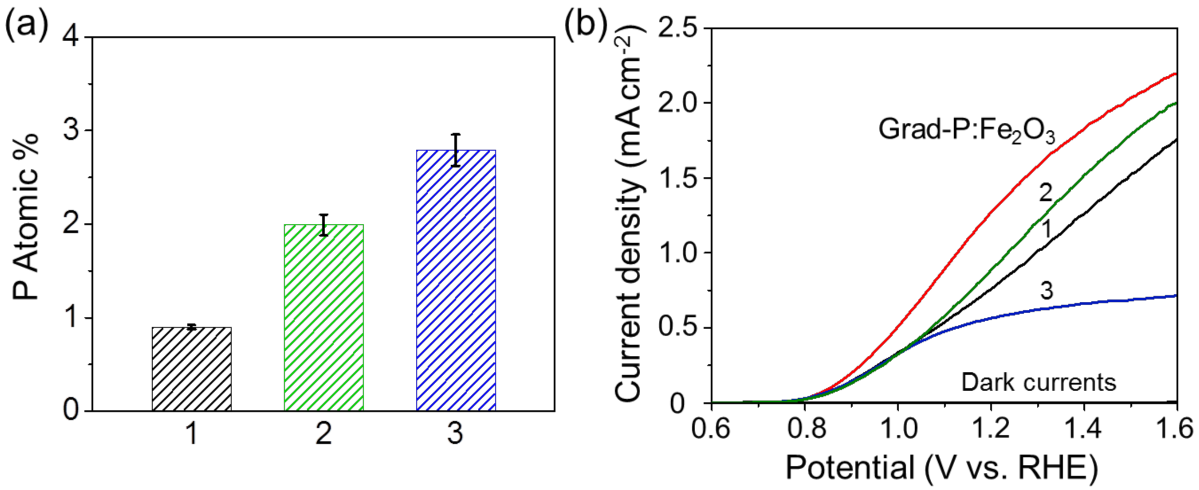

Fig. S8. The comparison of photocurrent values of 10 repeated photoanodes at 1.23 V (vs. RHE) under AM 1.5G illumination with standard deviation marked as error bars. (1: bare Fe_2O_3, 2: homo-P:Fe_2O_3, 3: grad-P:Fe_2O_3, 4: homo-P:Fe_2O_3/Co-Pi, 5: grad-P:Fe_2O_3/Co-Pi)

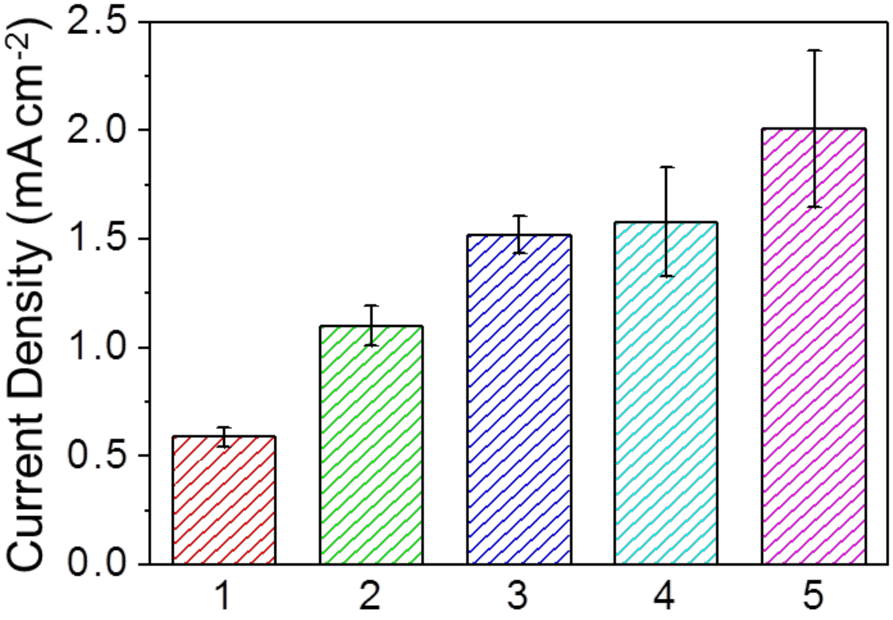

The lower performance of some grad-P:Fe_2O_3/Co-Pi samples compared with homo-P:Fe_2O_3/Co-Pi is likely due to the instability for the Co-Pi cocatalyst.

Fig. S9. (a) Schematic illustration of the testing electrode assemblies for the solid-state I-V curves of the electrodes. (b) Solid-state I-V characteristics of the representative electrodes. (1: F:SnO_2, 2: bare Fe_2O_3, 3: grad-P:Fe_2O_3, 4: homo-P:Fe_2O_3)

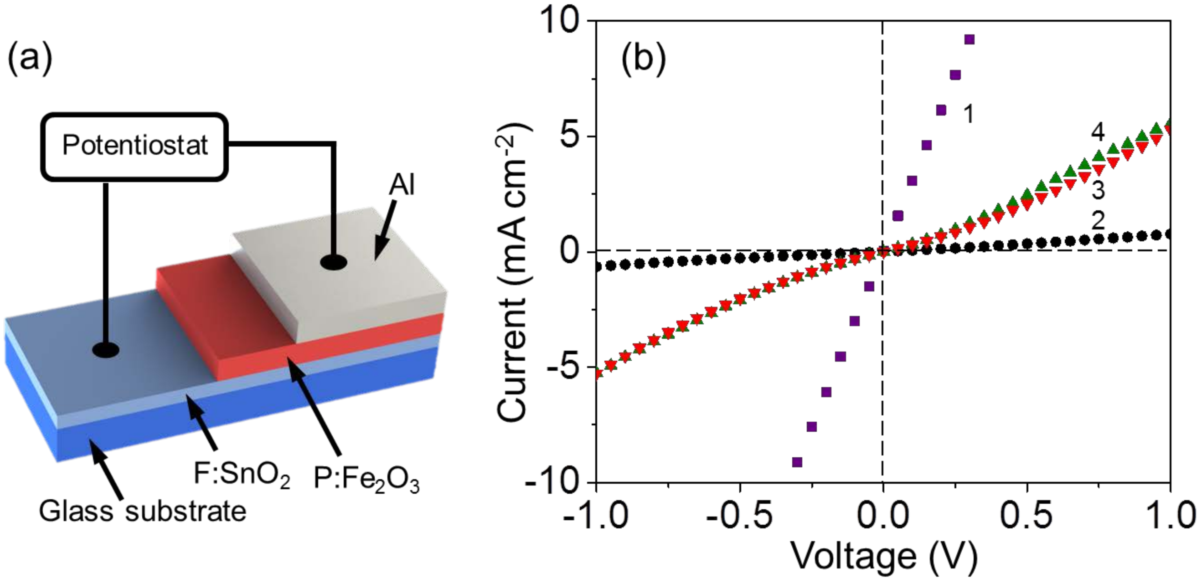

Table S1. The resistivity for the conductive FTO layers in various thermal treatment conditions.

Annealing condition	FTO [a] no annealing	500 °C for 2 h	750 °C for 10 min	750 °C for 30 min
Resistivity /10^{-4} Ω cm	6.13 ± 0.33	6.74 ± 0.45	8.70 ± 0.71	9.25 ± 0.14

[a] The resistivity of FTO measured by vendor (Wuhan Jinge-Solar Energy Technology Co., Ltd) is 5.60 × 10^{-4} Ω cm.

Table S2. Fitting results of the EIS of the bare Fe_2O_3, homo-P:Fe_2O_3 and grad-P:Fe_2O_3 photoanodes.

Samples	R_s/Ω	R_{ct}/Ω	R_{trap}/Ω
Bare Fe_2O_3	45.47 ± 0.59	213.1 ± 7.3	939.2 ± 35.7
Homo-Fe_2O_3	52.37 ± 0.48	111.2 ± 2.2	141.3 ± 3.6
Grad-Fe_2O_3	51.05 ± 0.46	105.3 ± 2.3	135.6 ± 3.7

Continued

Samples	C_{bulk}/μF	C_{trap}/μF	Chi-squared
Bare Fe_2O_3	0.009 ± 0.0003	0.23 ± 0.01	0.0306
Homo-Fe_2O_3	0.015 ± 0.0005	0.38 ± 0.02	0.0124
Grad-Fe_2O_3	0.016 ± 0.0005	0.40 ± 0.03	0.0137
